# Harnessing pre-trained models for accurate prediction of protein-ligand binding affinity

**DOI:** 10.1186/s12859-025-06064-w

**Published:** 2025-02-17

**Authors:** Jiashan Li, Xinqi Gong

**Affiliations:** Institute for Mathematical Sciences, School of Mathematics, Renmin University of China, 59 Zhongguancun Street, Beijing, 100872 China

**Keywords:** Binding affinity, Binding site prediction, Molecular representation, Molecular pre-training

## Abstract

****Background**:**

The binding between proteins and ligands plays a crucial role in the field of drug discovery. However, this area currently faces numerous challenges. On one hand, existing methods are constrained by the limited availability of labeled data, often performing inadequately when addressing complex protein-ligand interactions. On the other hand, many models struggle to effectively capture the flexible variations and relative spatial relationships between proteins and ligands. These issues not only significantly hinder the advancement of protein-ligand binding research but also adversely affect the accuracy and efficiency of drug discovery. Therefore, in response to these challenges, our study aims to enhance predictive capabilities through innovative approaches, providing more reliable support for drug discovery efforts.

****Methods**:**

This study leverages a pre-trained model with spatial awareness to enhance the prediction of protein-ligand binding affinity. By perturbing the structures of small molecules in a manner consistent with physical constraints and employing self-supervised tasks, we improve the representation of small molecule structures, allowing for better adaptation to affinity predictions. Meanwhile, our approach enables the identification of potential binding sites on proteins.

****Results**:**

Our model demonstrates a significantly higher correlation coefficient in binding affinity predictions. Extensive evaluation on the PDBBind v2019 refined set, CASF, and Merck FEP benchmarks confirms the model’s robustness and strong generalization across diverse datasets. Additionally, the model achieves over 95% in classification ROC for binding site identification, underscoring its high accuracy in pinpointing protein-ligand interaction regions.

****Conclusion**:**

This research presents a novel approach that not only enhances the accuracy of binding affinity predictions but also facilitates the identification of binding sites, showcasing the potential of pre-trained models in computational drug design. Data and code are available at https://github.com/MIALAB-RUC/SableBind.

## Background

Protein-ligand binding affinity prediction plays a crucial role in drug discovery and development. Accurate estimation of the binding affinity between protein and ligand is essential for identifying potential drug candidates and optimizing their therapeutic efficacy. Traditional experimental methods for measuring binding affinities are time-consuming, expensive, and often limited by the availability of target proteins [1, 2]. Consequently, computational approaches have emerged as valuable tools to predict binding affinities, offering faster and more cost-effective alternatives.

In the early stages of computational biology, the exploration of protein-ligand binding affinity already begins. During this period, Quantitative Structure-Activity Relationship (QSAR) models play a central and dominant role in the field of drug design [3, 4]. Empirical scoring functions are commonly employed to predict the binding affinity between protein targets and their ligands, with the goal of enhancing the success rate of drug design and reducing the cost of drug screening [5]. Widely used scoring functions, such as X-score [6] and Glide score [7], exemplify this approach. However, these methods suffer from significant limitations, including high target dependency and poor sensitivity to analogs. This is primarily due to their reliance on simplistic mathematical models, in which the fine-grained differences in molecular interactions are often overlooked, and the flexibility of proteins and ligands cannot be effectively accounted for. As research progresses, Molecular Dynamics (MD) simulations gradually emerge as a more refined tool [8]. By solving the classical equations of motion, MD simulations model the time-evolution of molecules, thereby capturing important details of the dynamic process. This enables a more accurate prediction of the thermodynamic characteristics of protein-ligand binding, overcoming the limitations of traditional methods.

With the advent of deep learning and large-scale pre-trained models, protein-ligand binding affinity prediction has seen significant advancements. Techniques such as convolutional neural networks (CNNs) and graph neural networks (GNNs) prove highly effective in capturing the intricate spatial and topological features of proteins and ligands. These methods excel in learning from large-scale datasets, enabling improved accuracy and generalization in modeling complex molecular interactions. In CNN-based approaches, methods like KDEEP [9] leverage 3D voxelized representations to encode both proteins and ligands, capturing crucial geometric and chemical properties that contribute to binding affinity. Pafnucy [10] also employs a deep convolutional architecture, processing 3D molecular complexes to extract spatial features across multiple convolutional layers, thereby enhancing the prediction of binding affinity. Additionally, RosENet [11] integrates molecular mechanics with deep learning, using a series of 3D CNNs to represent proteins and ligands and combining geometric and energetic information for more accurate predictions. On the other hand, GNN-based approaches model molecular structures as graphs, where atoms are represented as nodes and bonds or interactions as edges. This representation naturally captures the complex relationships within molecules. PotentialNet [12] uses graph convolutions to effectively learn both intramolecular and intermolecular interactions, making it a powerful tool for binding affinity prediction. RTMScore [13] further refines this approach by incorporating residue-atom distance likelihood potentials and leveraging graph transformers to achieve state-of-the-art performance. Similarly, PIGNet [14] employs gated graph attention networks (GATs) to iteratively update node features, considering both covalent and intermolecular interactions to predict binding affinity robustly.

Another significant advantage of deep learning models lies in the use of pre-trained models. Pre-trained models can learn complex relationships between proteins and ligands from large datasets and apply this knowledge to specific downstream tasks through transfer learning. Pre-trained models for small molecules have also demonstrated great potential. These models leverage different levels of molecular representation, including 1D molecular sequences [15–17], 2D graph representations [18, 19], and 3D structures [20]. By incorporating various forms of molecular representation, pre-trained models capture diverse molecular features and can be applied in multiple contexts, such as property prediction [21–23], drug discovery [24, 25], and virtual screening [26]. 3D structural information is particularly advantageous for capturing real molecular conformations and interactions, especially in tasks involving protein-ligand binding. Models like Uni-Mol [27], BindNet [28], and Frad [29] have demonstrated widespread applications in protein-ligand prediction. Pre-trained models not only address the traditional reliance on limited experimental datasets but also generate more robust and comprehensive protein and ligand representations, significantly improving the accuracy of binding affinity predictions.

In summary, while significant progress has been made in protein-ligand binding affinity prediction, several challenges remain. Current models still struggle with the scarcity of experimental data, especially for novel compounds and rare protein targets. Pre-trained models offer a promising solution by leveraging large-scale data to generate more generalizable molecular representations.

This paper presents a novel approach for predicting protein-ligand binding affinity based on pre-trained models, aimed at capturing the intricate molecular interactions and structural characteristics that influence binding affinity, using only the overall protein structure without requiring specific binding pocket information. Specifically, we develop a pre-trained model tailored for molecules, which constructs a self-supervised task that accurately models the three-dimensional structure and distance information of these molecules, adhering to fundamental molecular physics principles. By integrating one-dimensional sequence information with three-dimensional structural data, our method not only achieves precise predictions of binding affinity but also provides profound insights into protein binding sites, thereby offering new perspectives for drug design and molecular screening. We employ an innovative self-supervised learning strategy that enables the model to effectively capture diverse molecular features during the training process. Through extensive experimental evaluations on diverse benchmark datasets, we demonstrate the significant performance improvements of our pre-trained model over traditional computational methods.

## Methods

### Problem formulation

Given a protein *P* and a ligand *L*, we define:

- Let $P = \{ r_1, r_2, \ldots , r_n \}$ be the set of residues in the protein, where $r_i \in \mathbb {R}^{n \times 20}$, *n* is the total number of residues. The atomic coordinates of the residue are represented as $\textbf{x}_{i,k} \in \mathbb {R}^3$ where *k* denotes the index of the atoms within the residue.
- Let $L = \{ a_1, a_2, \ldots , a_m \}$ be the set of atoms in the ligand, where *m* is the total number of atoms. Each atom $a_j$ is represented by one of the 26 predefined common atomic types. The spatial coordinates of the atom are denoted by $\textbf{x}_j \in \mathbb {R}^3$.

**Fig. 1:**
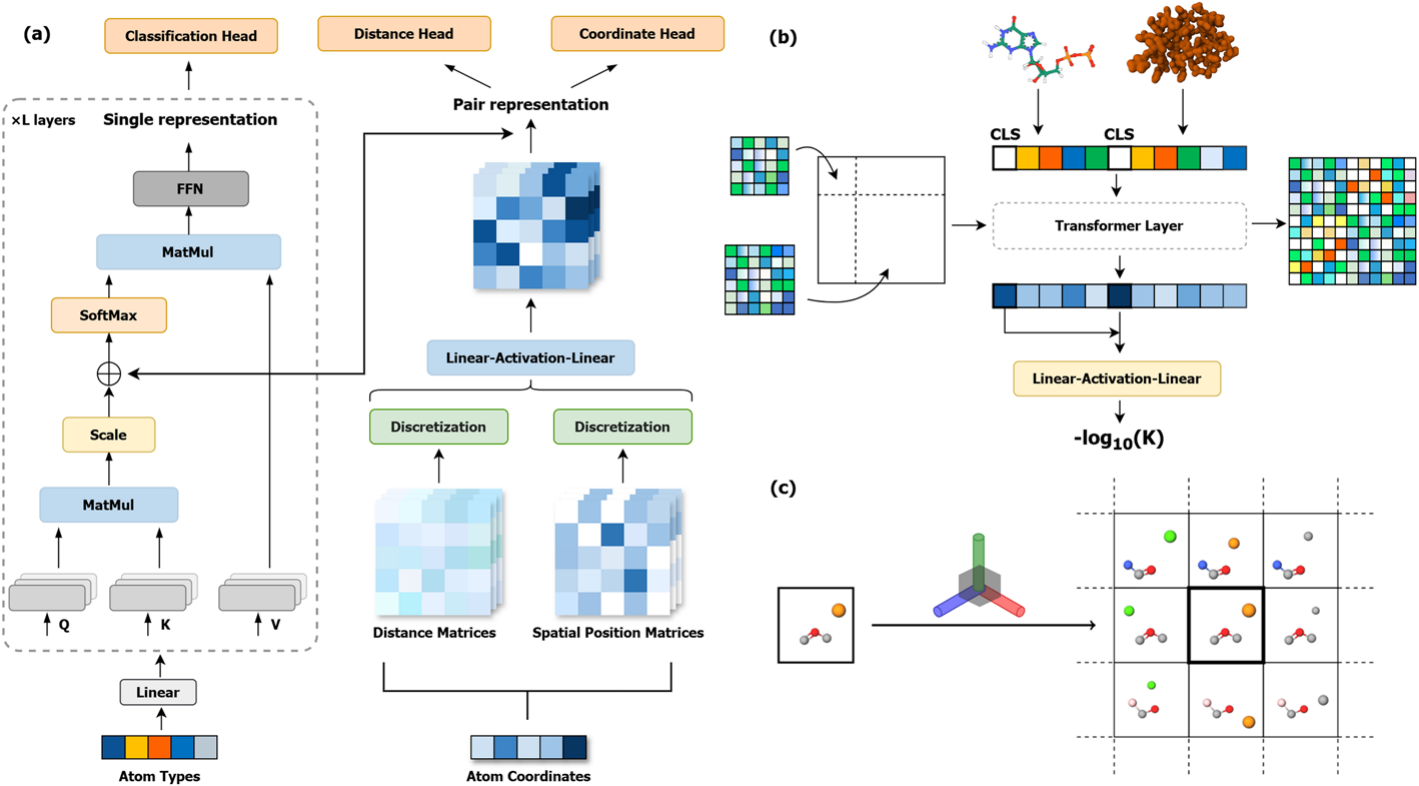
Overview. **a** Pre-training phase for ligands: Atom types are encoded via a linear layer as input to the transformer, while atomic coordinates are represented using a distance matrix and a spatial position matrix, creating an initial pair representation that serves as attention bias. Pre-training involves three self-supervised tasks: classification head, distance head, and coordinate head. **b** Affinity prediction for unknown protein-ligand complexes: Initial representations for proteins and ligands are derived from the pre-trained model and concatenated to form the initial representation of the complex, which lacks relative positional information between the protein and ligand. This representation is updated through transformer layers to predict binding affinity values. **c** Generation of spatial position representations from atomic coordinates: The spatial Cartesian coordinate system for a given atom is defined by its neighboring atoms ($i-1$, *i*, $i+1$), allowing for the determination of the spatial positions of all other atoms

Our objective is to learn a model $f: (P, L) \rightarrow y$ that accurately predicts the protein-ligand binding affinity *y* (Fig. 1b).

### Molecular representation

#### Structure encoder

The spatial coordinates of the atoms in the ligand are first converted into a distance matrix $D$ and a spatial position matrix $P$. The calculation of the distance matrix is defined as:

1 $$\begin{aligned} d_{ij} = \Vert \textbf{x}_i - \textbf{x}_j \Vert _2 \end{aligned}$$

where $\textbf{x}_i$ and $\textbf{x}_j$ denote the coordinates of atoms $i$ and $j$, respectively.

Next, we construct the spatial position matrix $P$ to represent the relative positional relationships among the atoms.  As shown in Fig. 1c, this process begins by defining a local coordinate system for each atom based on its neighboring atoms. Specifically, we derive the following basis vectors from the coordinates of neighboring atoms:

2 $$\begin{aligned} \textbf{v}_1&= \textbf{x}_{i} - \textbf{x}_{i-1}, \\ \textbf{v}_2&= \textbf{x}_{i+1} - \textbf{x}_{i}, \\ \textbf{v}_3&= \textbf{x}_{i+1} - \textbf{x}_{i-1}. \end{aligned}$$

We then apply the Gram-Schmidt process to orthogonalize these vectors:

3 $$\begin{aligned} \textbf{u}_1&= \textbf{v}_1, \\ \textbf{u}_2&= \textbf{v}_2 - \frac{(\textbf{v}_2 \cdot \textbf{u}_1)}{(\textbf{u}_1 \cdot \textbf{u}_1)} \textbf{u}_1. \\ \textbf{u}_3&= \textbf{v}_3 - \frac{(\textbf{v}_3 \cdot \textbf{u}_1)}{(\textbf{u}_1 \cdot \textbf{u}_1)} \textbf{u}_1 - \frac{(\textbf{v}_3 \cdot \textbf{u}_2)}{(\textbf{u}_2 \cdot \textbf{u}_2)} \textbf{u}_2. \end{aligned}$$

Using these orthogonalized vectors, we establish a coordinate system that effectively represents the position of atom $j$ within this frame. The resulting element $P_{ij}$ of the spatial position matrix thus captures the relative spatial positioning of the atoms in the ligand.

After discretizing the aforementioned matrices, we form the initial pair representation $\textbf{z}^{(0)}$ through a combination of linear transformation and activation functions:

4 $$\begin{aligned} \textbf{z}^{(0)} = \text {Linear}\left( \sigma \left( \text {Linear}\left( \textbf{D} \oplus \textbf{P} \right) \right) \right) \end{aligned}$$

where $\oplus$ denotes the concatenation operation and $\sigma$ is the activation function.

#### Backbone network

 We employ a standard Transformer architecture as the backbone network for our pre-training model, as shown in Fig. 1a. The input to the Transformer consists of a sequence of atomic types represented by $Ea_i$, which are projected through a linear layer. Additionally, we introduce a special token $[\text {CLS}]$, whose coordinates are set to the centroid of all atomic coordinates. The input vector is then fed into the Transformer.

The Transformer consists of *L* layers. For each layer *l*, the self-attention mechanism computes the attention weights as follows:

5 $$\begin{aligned} \text {Attention}(Q^{(l)}, K^{(l)}, V^{(l)}) = \text {softmax}\left( \frac{Q^{(l)}K^{(l)T}}{\sqrt{d_k}} + z^{(l-1)}\right) V^{(l)} \end{aligned}$$

where $Q^{(l)}$, $K^{(l)}$, and $V^{(l)}$ are the query, key, and value matrices for layer *l*, respectively. Here, $z^{(l)}$ is a bias term capturing pairwise relationships and is updated at each layer as follows:

6 $$\begin{aligned} z^{(l)} = z^{(l-1)} + \text {Attention}(Q^{(l-1)}, K^{(l-1)}, V^{(l-1)}) \end{aligned}$$

Ultimately, the model produces two types of representations from the output of the final layer: a single representation *s* and a pair representation *z*.

#### Self-supervised learning task

 During the training process, we do not introduce additional labels; instead, we employ a self-supervised learning approach through random masking of atomic types and the application of noise addition and denoising to atomic coordinates. Each training iteration involves randomly selecting varying proportions of atoms for the masking operation to enhance the model’s generalization capabilities. We utilize a noise addition method based on atomic potential energy gradients for perturbing atomic coordinates. The gradient of atomic potential energy represents the rate of change of potential energy in each direction, thereby reflecting the forces acting on the atoms. By adding noise along the direction of the potential energy gradient and adjusting the amplitude of the noise based on the magnitudes of interatomic interactions, our noise addition method is more consistent with physical laws, thereby avoiding non-physical variations introduced by simple random noise. This strategy not only enhances the physical consistency of the model but also effectively improves its learning capacity.

The potential energy *V* between two atoms can be described by the Lennard–Jones potential function:

7 $$\begin{aligned} V(r) = 4\epsilon \left[ \left( \frac{\sigma }{r}\right) ^{12}-\left( \frac{\sigma }{r}\right) ^{6}\right] \end{aligned}$$

where *r* is the distance between atoms, and $\epsilon$ and $\sigma$ are parameters related to the types of atoms.

To calculate the gradient of the potential energy with respect to the atomic coordinates $\textbf{x}$, we use the finite difference method:

8 $$\begin{aligned} \frac{\partial V}{\partial \textbf{x}_i} \approx \frac{V(\textbf{x} + h\textbf{e}_i) - V(\textbf{x} - h\textbf{e}_i)}{2h} \end{aligned}$$

where *h* is a small perturbation and $\textbf{e}_i$ is the unit vector in the direction of the *i*-th coordinate.

Based on the calculated gradient $\nabla V$, we introduce noise into the atomic coordinates $\textbf{x}$ as follows:

9 $$\begin{aligned} \textbf{x}_{\text {noisy}} = \textbf{x} + \eta \cdot \frac{\nabla V}{\Vert \nabla V\Vert } + \xi \end{aligned}$$

where $\eta$ represents a scaled perturbation based on the gradient magnitude, and $\xi$ is a small random noise term added to increase variability.

The denoising process utilizes a classification head to predict the probabilities of the atomic types, a distance head to recover the distance matrix, and a coordinate head to denoise the atomic coordinates.

### Protein representation and integration

In this section, we outline the process of obtaining representations for proteins and their integration with ligands. First, we derive the single representation $s_{\text {ligand}}$ and pair representation $z_{\text {ligand}}$ for the ligand from the pretrained model designed for small molecules. The single representation of the ligand is defined as:

10 $$\begin{aligned} s_{\text {ligand}} = [h_{L,[CLS]}, h_{L,1}, h_{L,2}, \ldots , h_{L,m}] \end{aligned}$$

where *m* is the number of atoms in the ligand.

Next, we encode the sequence and structural information of the protein *P* using $\mathcal {S}$able [30], resulting in the protein’s single representation $s_{\text {protein}}$ and pair representation $z_{\text {protein}}$:

11 $$\begin{aligned} s_{\text {protein}}, z_{\text {protein}} = \text {Sable}(P) \end{aligned}$$

The single representation of the protein is similarly defined as:

12 $$\begin{aligned} s_{\text {protein}} = [h_{P,[CLS]}, h_{P,1}, h_{P,2}, \ldots , h_{P,n}] \end{aligned}$$

where *n* is the number of residues in the protein.

To generate the representation of the protein-ligand complex, we concatenate the representation of the ligand and protein:

13 $$\begin{aligned} s_{\text {complex}} = [h_{L,[CLS]}, h_{L,1}, h_{L,2}, \ldots , h_{L,m}, h_{P,[CLS]}, h_{P,1}, h_{P,2}, \ldots , h_{P,n}] \end{aligned}$$

The pair representation for the complex $z_{\text {complex}}$ is structured as follows:

14 $$\begin{aligned} z_{\text {complex}} = \begin{bmatrix} z_{\text {ligand}} & 0 \\ 0 & z_{\text {protein}} \end{bmatrix} \end{aligned}$$

In this matrix, $z_{\text {ligand}}$ encodes the interactions among the ligand’s atoms, while $z_{\text {protein}}$ captures the interactions among the protein’s residues. The zeroes in the pair representation $z_{\text {complex}}$ serve as padding, representing the unknown interactions between the ligand and the protein chain. This padding ensures that the dimensions of the concatenated matrix align appropriately while indicating that there is no direct information available regarding the interactions between the ligand and the protein residues.

### Model architecture

The representations of the ligand and protein, denoted as $s_{\text {complex}}$ and $z_{\text {complex}}$, are used as the input to our protein-ligand affinity prediction network. This process is similar to the ligand pretraining backbone network. In this model, the single representation $s_{\text {complex}}$ is fed into a standard Transformer pipeline, while the pair representation $z_{\text {complex}}$ is incorporated as an attention bias into the attention mechanism.

The Transformer updates both $s_{\text {complex}}$ and $z_{\text {complex}}$ through multiple layers, capturing interactions between the ligand and protein representations:

15 $$\begin{aligned} s_{\text {complex}}^{\text {update}}, z_{\text {complex}}^{\text {update}} = \text {Transformer}(s_{\text {complex}}, z_{\text {complex}}) \end{aligned}$$

The updated $s_{\text {complex}}^{\text {update}}$ is passed through several Transformer layers to further capture the intricate ligand-protein interactions.

Subsequently, we extract the [*CLS*] tokens from both the ligand and protein, representing their global embeddings. These embeddings are concatenated as follows:

16 $$\begin{aligned} h_{\text {concat}} = [\text {CLS}_{\text {ligand}}, \text {CLS}_{\text {protein}}] \end{aligned}$$

The concatenated vector is then passed through a fully connected feedforward network with an activation function to predict the binding affinity:

17 $$\begin{aligned} \hat{y} = \text {Linear} \left( \sigma \left( \text {Linear}(h_{\text {concat}}) \right) \right) \end{aligned}$$

where $\hat{y}$ represents the predicted binding affinity, and $\sigma$ is the activation function. The training objective is to minimize the Mean Squared Error (MSE) between the predicted and true binding affinities:

18 $$\begin{aligned} \mathcal {L}_{\text {MSE}} = \frac{1}{S} \sum _{i=1}^{S} (\hat{y}_i - y_i)^2 \end{aligned}$$

where $S$ is the total number of training samples, $\hat{y}_i$ is the predicted binding affinity for the $i$-th sample, and $y_i$ is the corresponding ground truth binding affinity.

We treat the binding site prediction as a binary classification task, where residues are classified as binding sites if their distance to any ligand atom is below a predefined threshold. To achieve this, we extract the protein representation $s_{\text {protein}}^{\text {update}}$ from the updated complex representation $s_{\text {complex}}^{\text {update}}$.

The classification is performed using the following equation:

19 $$\begin{aligned} \hat{p} = \text {Linear} \left( \sigma \left( \text {Linear}(s_{\text {protein}}^{\text {update}}) \right) \right) \end{aligned}$$

Here, $\hat{p}$ represents the predicted probability for each residue in the protein being a binding site. To train the model, we use a binary cross-entropy loss function:

20 $$\begin{aligned} \mathcal {L}_{\text {BCE}} = - \frac{1}{N} \sum _{i=1}^{N} \left[ y_i \log (\hat{p}) + (1 - y_i) \log (1 - \hat{p}) \right] \end{aligned}$$

where $y_i$ is the true label for residue $i$ (0 for non-binding, 1 for binding), $\hat{p}$ is the predicted probability for each residue, and $N$ is the total number of residues.

## Results

### Datasets

#### Ligand pre-training datasets

The ligand pre-training dataset used in this study is sourced from the Uni-Mol  [27] project, constructed from multiple public repositories. After normalization and deduplication processes, the dataset comprises approximately 19 million unique molecules. Each molecule features 10 distinct conformations, and during training, a random conformation is selected for each molecule to enhance variability.

#### Protein-Ligand binding affinity datasets

The protein-ligand binding affinity datasets used in this study are primarily derived from the PDBBind Database [31, 32], which contains protein-ligand complex structures determined by X-ray crystallography. The binding affinities provided by PDBBind are experimentally measured and presented in terms of inhibition constant ($K_i$), dissociation constant ($K_d$), or half-maximal inhibitory concentration (IC50), all in molar units. We employ the pK metric, defined as the negative logarithm of the binding affinity, which is consistent with approaches used in previous studies. Specifically, pK is calculated as follows:

21 $$\begin{aligned} \text {pK} = -\log _{10} \left( \frac{K_d \hspace{5.0pt}\text {or} \hspace{5.0pt}K_i}{1 \times 10^9} \right) \end{aligned}$$

PDBBind v2019 Refined Set consists of 4852 complexes, which are carefully curated based on several quality criteria, including a resolution of $\le 2.5$   Å, an R-factor $\le 0.25$, and the absence of steric clashes or covalent bonds. Complexes with unreliable ligand binding data are excluded. To ensure the reliability of the evaluation and to avoid overfitting, we implement a protein sequence-based splitting strategy, which results in two distinct partitioning standards: one with a 30% sequence identity threshold and another with a 60% sequence identity threshold. These partitions are designed to prevent homologous proteins from appearing in both the training and test sets. Following the Atom3D [33] partitioning method, the dataset with a 30% sequence identity threshold contains 3507 proteins in the training set, 466 proteins in the validation set, and 490 proteins in the test set. Similarly, using the 60% sequence identity threshold, the training set consists of 3678 proteins, the validation set contains 460 proteins, and the test set includes 460 proteins. This partitioning strategy enables us to evaluate the model’s performance in a way that reflects its generalization ability across different protein families.

To improve the reliability and reproducibility of model evaluation, we implement ten-fold cross-validation on both the LBA30 and LBA60 datasets. Taking the LBA30 dataset as an example, we first randomly divide the entire dataset (including the training, validation, and test sets) into 10 subsets of roughly equal size. In each round of cross-validation, we sequentially select one subset as the test set, another subset as the validation set, and combine the remaining 8 subsets to form the training set. By repeating this process, we train and evaluate the model multiple times, enabling us to assess the model’s performance stability and generalization ability across different data splits.

PDBBind v2020 General Set consists of 19,443 complexes. Following the data splitting strategy from *TopoFormer*, after excluding CASF-2007, CASF-2013, CASF-2016, and PDBBind 2016 core set, the remaining 18,904 complexes are used as the training set for the model.

CASF Benchmark Dataset is an open-access benchmark dataset for evaluating the performance of scoring functions. We select CASF-2007, CASF-2013 and CASF-2016 as benchmark test sets, which contain 195, 195 and 285 protein-ligand complexes, respectively. These complexes feature high-quality crystal structures and reliable binding constants.

Merck FEP Benchmark Dataset differs significantly from PDBBind in terms of its distribution. Originally developed to assess free energy prediction models, this dataset focuses on evaluating the model’s ability to rank the relative binding free energies of ligands that bind to the same target and share a similar scaffold. The dataset includes 264 active ligands across 8 drug-related targets, with binding affinity data curated from relevant literature. We convert the binding free energy data into binding affinity values using the following formula:

22 $$\begin{aligned} \Delta G = -RT \ln K \end{aligned}$$

where *R* is the gas constant, *T* is the temperature in Kelvin, and *K* is the dissociation constant. This dataset serves as an additional test set alongside the CASF benchmark dataset for evaluation of the PDBBind v2020 General Set.

#### Protein-Ligand binding site datasets

The dataset for protein-ligand binding site prediction is derived from the PDBBind v2019 Refined Set, utilizing both the LBA30 and LBA60 datasets. Ten-fold cross-validation is applied to ensure robust evaluation and reproducibility of the model performance. To facilitate binding site prediction, a residue is defined as a binding site if any heavy atom in the protein residue is within a predefined cut-off distance from the ligand. Specifically, the cut-off distance is set to 6Åin the main experiments. Additionally, ablation studies are conducted using cut-offs of 4Åand 8Åto assess the sensitivity of model performance to varying proximity thresholds.

### Evaluation metrics

To comprehensively assess the performance of our model, we employ several evaluation metrics, including the Root Mean Square Error (RMSE), Pearson correlation coefficient, Spearman correlation coefficient, and Area Under the Receiver Operating Characteristic Curve (AUC). The RMSE, Pearson, and Spearman coefficients are essential for quantifying the predictive accuracy and the relationship between predicted and actual values in regression tasks, while AUC evaluates the model’s ability to predict binding sites.

The Root Mean Square Error (RMSE) is defined as follows:

23 $$\begin{aligned} \text {RMSE} = \sqrt{\frac{1}{n} \sum _{i=1}^{n} (y_i - \hat{y}_i)^2} \end{aligned}$$

where $y_i$ represents the actual values, $\hat{y}_i$ denotes the predicted values, and $n$ is the total number of samples.

The Pearson correlation coefficient is calculated as:

24 $$\begin{aligned} r = \frac{\sum _{i=1}^{n} (y_i - \bar{y})(\hat{y}_i - \bar{\hat{y}})}{\sqrt{\sum _{i=1}^{n} (y_i - \bar{y})^2} \sqrt{\sum _{i=1}^{n} (\hat{y}_i - \bar{\hat{y}})^2}} \end{aligned}$$

where $\bar{y}$ and $\bar{\hat{y}}$ are the mean values of the actual and predicted outputs, respectively.

Finally, the Spearman correlation coefficient is given by:

25 $$\begin{aligned} \rho = 1 - \frac{6 \sum d_i^2}{n(n^2 - 1)} \end{aligned}$$

where $d_i$ is the difference between the ranks of the actual and predicted values, and $n$ is the number of observations.

The AUC is calculated as follows:

26 $$\begin{aligned} \text {AUC} = \int _{0}^{1} \text {TPR}(x) \, dx \end{aligned}$$

where TPR (True Positive Rate) is defined as:

27 $$\begin{aligned} \text {TPR} = \frac{\text {True Positives}}{\text {True Positives} + \text {False Negatives}} \end{aligned}$$

These metrics collectively provide a comprehensive evaluation of our model’s performance in both regression and classification tasks.

### Evaluation of model performance

#### Assessment on PDBBind v2019 refined set

To evaluate the performance of our model, we conduct experiments on datasets with different identity clustering thresholds from the refined set of PDBBind. We compare our method with a total of 11 methods in three categories: sequence-based, structure-based, and pre-trained methods. SableBind achieves state-of-the-art Pearson correlation coefficient and Spearman coefficient on the LBA60 dataset and outperforms sequence-based and structure-based methods in most metrics. While SableBind shows slightly inferior performance compared to BindNet on the LBA30 dataset, this is attributed to BindNet’s use of protein pocket data. This means that before predicting the numerical value of protein-ligand binding affinity, it already has prior knowledge of the binding site. In contrast, our model can predict protein-ligand binding affinity even when the binding site is unknown. Moreover, our model can provide insights into potential binding sites through analyzing various features and patterns without relying on pre-existing knowledge of specific binding locations.

To further assess the robustness of our model, we perform a ten-fold cross-validation on the LBA30 and LBA60 datasets to evaluate the impact of data partitioning on the results. For the LBA30 dataset, the cross-validation results show a lower standard deviation, indicating more consistent performance across different splits. Specifically, the standard deviation of Pearson’s correlation is 0.0617, RMSE is 0.0965, and Spearman’s correlation is 0.0597, suggesting that the model’s performance remains stable regardless of how the data is divided. For the LBA60 dataset, the standard deviation of Pearson’s correlation is 0.0449, RMSE is 0.0899, and Spearman’s correlation is 0.0465 in the ten-fold cross-validation. Compared to the LBA30 dataset, the results across different data splits are more consistent. However, the performance is noticeably lower than in the original specific data partitioning scenario. This is likely due to the random shuffling of data in the ten-fold cross-validation, which may result in training and testing sets that no longer contain pairs with high sequence identity. This reduces the model’s ability to leverage sequence similarity. Therefore, in the cross-validation setup, the results for LBA30 and LBA60 become much closer, as the model relies more on other features beyond sequence similarity.

**Fig. 2:**
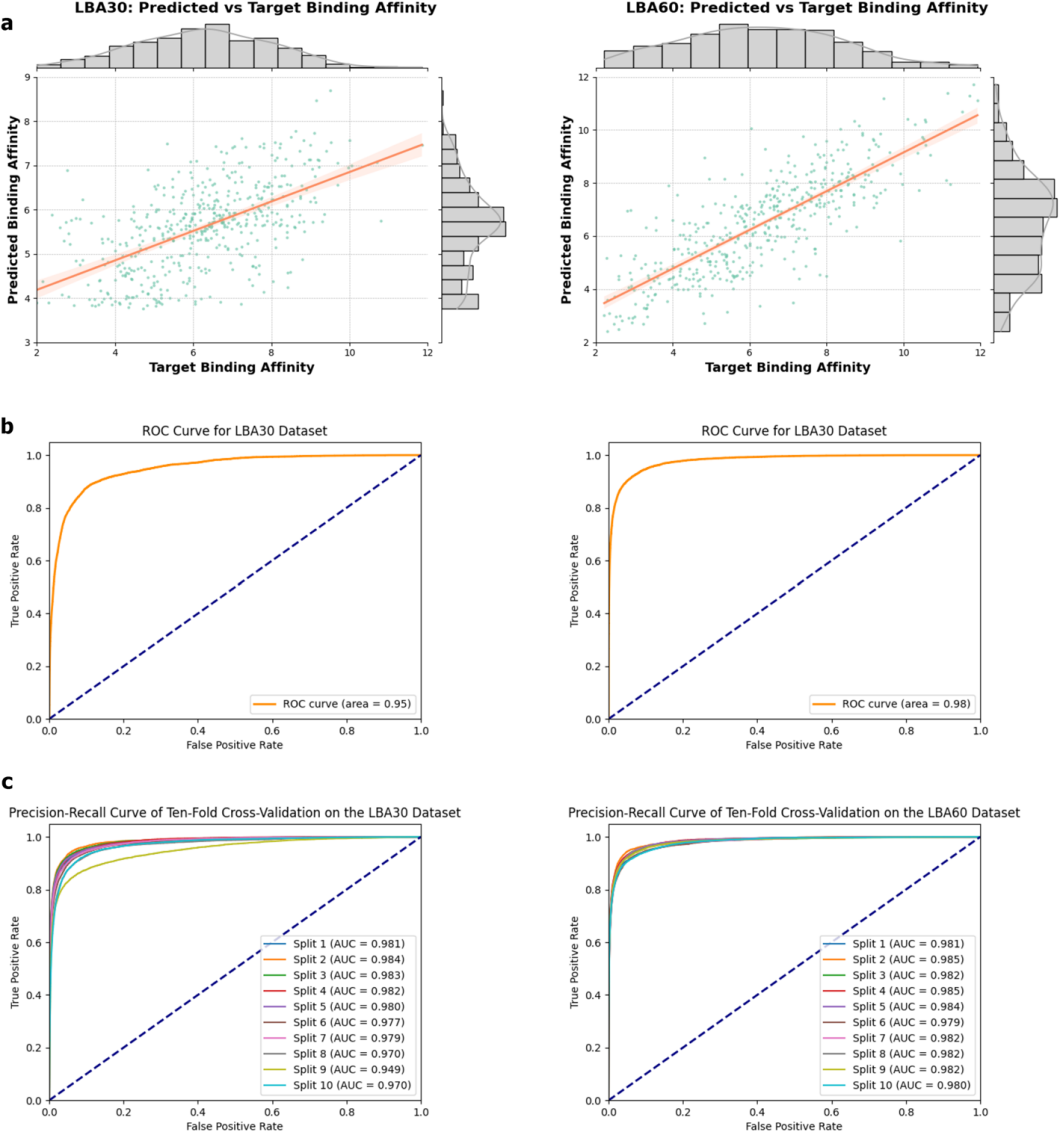
Overview of results from our binding affinity and binding site prediction analyses, comparing the LBA30 dataset on the left and the LBA60 dataset on the right. Panel **a** presents the marginal distribution histograms of predicted binding affinities, while panel **b** displays the Receiver Operating Characteristic (ROC) curves for protein-ligand binding site predictions. Panel **c** shows the results from ten-fold cross-validation on both LBA30 and LBA60 datasets

Despite these differences, the overall trend indicates that SableBind achieves high performance even in the absence of specific binding site knowledge and generalizes well across various data splits. The results are summarized in Table 1, and Fig. 2a presents the marginal distribution histograms of predicted versus true values, illustrating the consistency and accuracy of our method across different data subsets.

**Table 1:** Performance comparison of various methods on LBA dataset under different protein sequence identity split settings

Method	LBA30			LBA60		
	RMSE $\downarrow$	Pearson $\uparrow$	Spearman $\uparrow$	RMSE $\downarrow$	Pearson $\uparrow$	Spearman $\uparrow$
DeepDTA [34]	1.866	0.472	0.471	1.762	0.666	0.663
TAPE [35]	1.890	0.338	0.286	1.633	0.568	0.571
ProtTrans [36]	1.544	0.438	0.434	1.641	0.595	0.588
Atom3D-CNN [33]	1.416	0.550	0.553	1.621	0.608	0.615
Atom3D-ENN [33]	1.568	0.389	0.408	1.620	0.623	0.633
Atom3D-GNN [33]	1.601	0.545	0.533	1.408	0.743	0.743
Holoprot [37]	1.464	0.509	0.500	1.365	0.749	0.742
ProNet [38]	1.463	0.551	0.551	1.343	0.765	0.761
DeepAffinity [39]	1.893	0.415	0.426	–	–	–
SMT-DTA [40]	1.574	0.458	0.447	1.347	0.758	0.754
GeoSSL [41]	1.451	0.577	0.572	–	–	–
Uni-Mol [27]	1.434	0.565	0.540	1.357	0.753	0.750
BindNet [28]	**1.340**	**0.632**	**0.620**	**1.230**	0.793	0.788
SableBind	1.527	0.579	0.579	1.246	**0.802**	**0.798**
*Cross-Validation*	*1.581*	*0.616*	*0.620*	*1.562*	*0.630*	*0.632*

The methods are organized into three categories: sequence-based methods at the top, followed by structure-based methods, and finally pre-training approaches at the bottom. The best and second-best results are highlighted in **bold** and underlined, respectively. The results of SableBind-cross-validation are presented with a italic background, and are not included in the ranking for best results due to differing dataset partitioning

#### Assessment on CASF benchmark

CASF is a key benchmark in the field of protein-ligand interaction prediction. To evaluate the performance of SableBind and compare it with current state-of-the-art methods, we test it on the CASF-2007, CASF-2013, and CASF-2016 datasets.  The results, summarized in Table 2, provide a comprehensive comparison across these datasets, highlighting SableBind’s strengths and potential. While no method achieves perfect performance across all datasets and metrics, SableBind achieves SOTA in Spearman’s correlation on the CASF-2016 dataset and shows competitive results in other metrics, with a balanced overall performance.

**Table 2:** Performance comparison on CASF-2007, CASF-2013, and CASF-2016 datasets

Method	CASF-2007	CASF-2013	CASF-2016		
	Pearson $\uparrow$	Pearson $\uparrow$	RMSE $\downarrow$	Pearson $\uparrow$	Spearman $\uparrow$
AutodockVina [42]	–	0.54	–	0.604	0.528
Glide-SP [7]	0.343	0.452	–	0.513	0.419
Glide-XP [43]	0.457	0.277	–	0.467	0.257
ECIF [44]	–	–	1.169	**0.866**	–
CAPLA [45]	–	0.770	1.200	0.843	–
SVSBI [46]	–	–	–	0.832	–
KDEEP [9]	–	–	–	0.701	0.528
Pafnucy [10]	–	0.70	1.42	0.78	–
OnionNet-2 [47]	–	**0.821**	1.164	0.864	–
GenScore [48]	–	–	–	0.837	0.682
ConBAP [49]	–	–	**1.127**	0.864	0.719
PIGNet2 [50]	–	–	–	0.747	0.651
TopoFormer-Seq [51]	**0.836**	0.817	–	0.865	–
SableBind	0.826	0.787	1.205	0.832	**0.825**

The methods are divided into three categories: traditional scoring functions (top), sequence-based methods (middle), and structure-based methods (bottom). The best and second-best results are marked in **bold** and underlined, respectively

#### Assessment on Merck FEP benchmark

To more accurately assess the model’s generalization ability, we choose the Merck FEP Benchmark for testing, which has a distribution that is completely different from PDBBind. Unlike the high sequence similarity between the proteins in the CASF test set and those in the training set [59, 60], the Merck FEP Benchmark provides more diverse and complex protein-ligand interaction data, presenting a much more challenging task for the model. Thus, using the Merck FEP Benchmark enables a more effective evaluation of the model’s performance and robustness across different data distributions.  The results of the evaluation on the Merck FEP Benchmark are summarized in Table 3. On the 8 datasets covered by the Merck FEP Benchmark, SableBind achieves an average Pearson correlation coefficient of 0.479, ranking among the top methods, second only to GenScore, demonstrating strong competitiveness. In the eg5 dataset, SableBind achieves a Pearson correlation coefficient of 0.710, outperforming all other methods and highlighting its advantage in more complex tasks. In contrast, among the 14 baseline methods, 11 show negative correlations on certain datasets, meaning that these methods fail to effectively predict protein-ligand interactions in these scenarios and even exhibit trends contrary to the actual results, severely impacting their reliability and effectiveness in practical applications. SableBind, on the other hand, maintains positive correlations across all test sets, showcasing its stability and adaptability in various protein-ligand interaction prediction tasks. This indicates that SableBind has strong generalization ability, particularly excelling in handling complex and challenging prediction tasks.

**Table 3:** Performance comparison on the Merck FEP benchmark across 8 datasets, evaluated using Pearson correlation coefficients

Method	hif2a	pfkfb3	eg5	cdk8	shp2	syk	cmet	tnks2	Average
	(42)	(40)	(28)	(33)	(26)	(44)	(24)	(27)	(264)
X-Score [6]	0.224	0.430	− 0.316	0.406	− 0.030	0.689	0.531	0.669	0.325
Glide SP [7]	0.445	0.480	− 0.111	0.345	0.542	− 0.006	0.378	0.316	0.299
Glide XP [43]	0.410	0.513	0.017	0.617	0.490	0.124	0.165	0.582	0.365
AutoDock 4 [52]	0.376	0.530	− 0.397	0.629	0.609	0.544	0.324	0.558	0.397
Vina [42]	**0.493**	0.546	− 0.520	**0.849**	0.569	0.519	− 0.257	0.538	0.342
Vinardo [53]	0.371	0.515	− 0.475	0.782	0.490	0.379	− 0.359	0.305	0.251
SchNet [54]	0.103	0.242	0.361	0.334	0.078	0.281	0.233	0.231	0.232
Pafnucy [10]	0.224	0.430	− 0.316	0.406	− 0.030	**0.689**	0.531	**0.669**	0.325
PotentialNet [12]	0.247	0.344	0.416	0.168	0.029	0.173	0.283	− 0.001	0.207
GNN_DTI [55]	0.163	0.427	0.297	0.417	− 0.071	0.084	0.496	0.130	0.243
IGN [56]	0.207	0.292	0.022	0.362	− 0.200	0.098	0.670	0.077	0.192
$\Delta _{\text {Lin}\_\text {F9}}\text {XGB}$ [57]	0.480	0.603	− 0.099	0.826	**0.640**	0.103	0.077	0.458	0.386
GIGN [58]	0.303	0.427	0.183	0.290	0.371	0.012	0.35	− 0.038	0.238
GenScore [48]	0.455	**0.635**	0.293	0.693	0.489	− 0.001	**0.773**	0.598	**0.492**
ConBAP [49]	0.250	0.398	0.524	0.563	0.231	0.447	0.262	0.049	0.340
SableBind	0.428	0.485	**0.710**	0.555	0.440	0.189	0.631	0.391	0.479

The dataset sizes are indicated in parentheses. The methods are grouped into two categories: traditional scoring functions (top) and machine learning/deep learning methods (bottom). The best and second-best results are marked in **bold** and underlined, respectively

#### Assessment on bindind site prediction

**Fig. 3:**
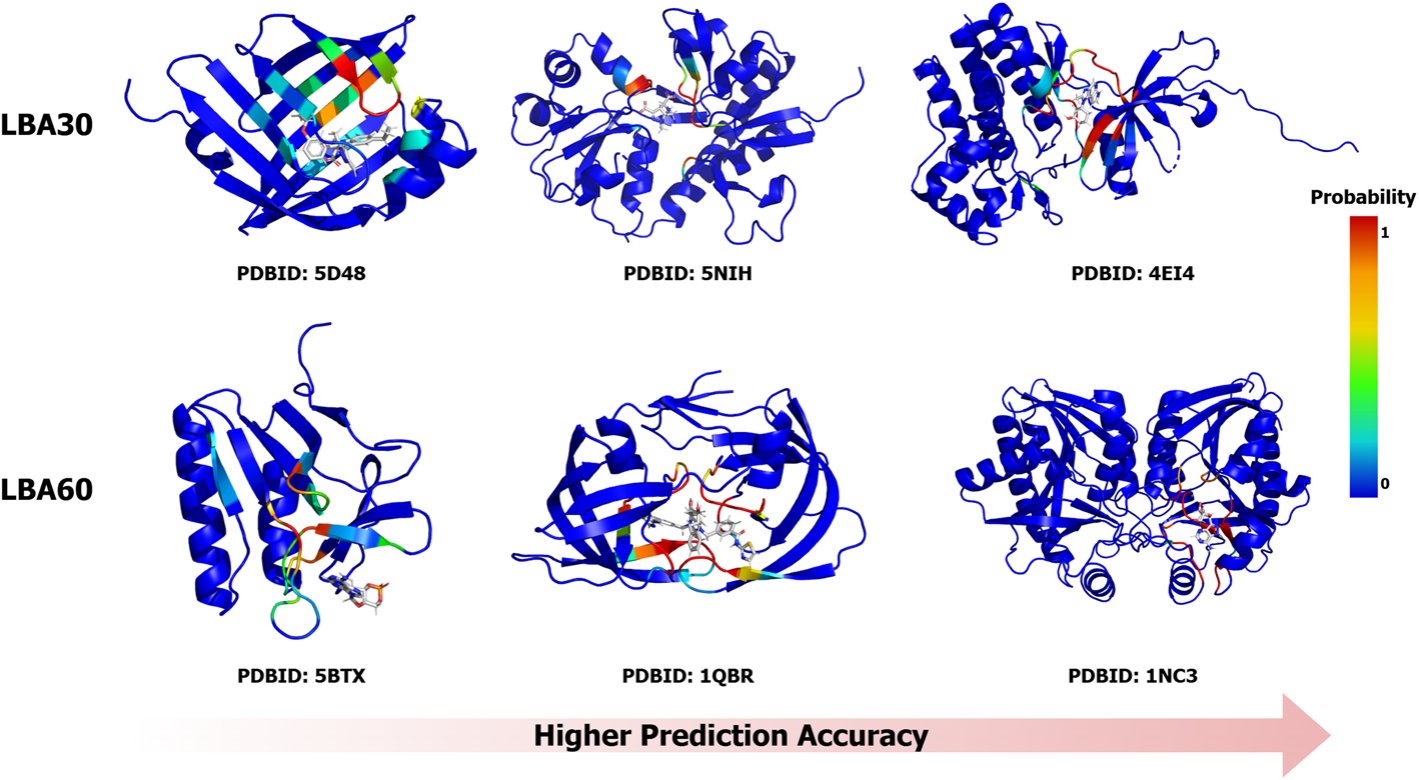
This case study illustrates the worst, intermediate, and best predictions of binding sites from two datasets. Protein structures are visualized in PyMOL and color-coded based on predicted probabilities, with red indicating higher confidence in binding site predictions and blue representing lower confidence. Ligands are rendered in gray

Furthermore, to provide insights into binding sites alongside binding affinity predictions, we test the model’s capability in predicting these sites. Our ROC analysis yields values exceeding 95% for both LBA30 and LBA60 datasets, indicating robust predictive performance for identifying binding sites. To illustrate the effectiveness of binding site predictions, we visualize the results in Fig. 3. For residues predicted with a probability greater than 0.5, we classify them as binding sites, calculating the accuracy as the ratio of correctly classified residues to the total sequence length. We select the worst, medium, and best predicted proteins for visualization. For LBA30, the accuracy of the worst prediction is 77.94%. Among the 490 proteins in the test set, 459 have an accuracy of over 90%. For LBA60, the accuracy of the worst prediction is 84.07%. Among the 452 proteins in the test set, 442 have an accuracy of over 90%. Our model can always provide reliable predictions and useful insights for understanding protein-ligand interactions.

To more comprehensively assess the stability and generalization ability of the model, we perform ten-fold cross-validation on the LBA30 and LBA60 datasets. This validation simulates different data splits to examine the model’s performance on diverse datasets. The results show that the performance across both datasets is similar, indicating that the model maintains stable prediction performance on datasets with varying levels of sequence consistency. It also demonstrates strong robustness and good adaptability to differences in dataset characteristics. Compared to the results obtained from the original splits, the model performs better in terms of average AUC and F1 scores during cross-validation. This indicates that the model can maintain high predictive accuracy across different data subsets, and its predictions exhibit lower variance, further confirming its strong generalization capability. Therefore, the model provides reliable binding site predictions across various data distributions, offering valuable support for protein-ligand interaction research. The results are summarized in Table 4 and Fig. 2c.

**Table 4:** Ten-fold cross-validation results for protein-ligand binding site prediction: Average AUC with standard deviation, and average F1 score with standard deviation for the LBA30 and LBA60 datasets

Dataset	Average AUC (Std)	Average F1 Score (Std)
LBA30	0.976 (0.010)	0.752 (0.031)
LBA60	0.982 (0.002)	0.777 (0.018)

### Experimental setup

The ligand pre-training phase utilizes a Transformer architecture comprising 15 layers, each equipped with 512 hidden units and 4 attention heads. The feed-forward network within each layer has a dimensionality of 2048, facilitating the model’s capacity to capture intricate relationships in the data. Pre-training is conducted over approximately 20 epochs, with a batch size of 128, allowing for efficient data processing and convergence.

For optimization, we employ the Adam optimizer with parameter settings of $\beta _1 = 0.9$ and $\beta _2 = 0.99$. The initial learning rate is set to $1 \times 10^{-4}$, accompanied by a warm-up period of 10,000 steps, followed by linear decay to stabilize training. Additionally, a weight decay of $1 \times 10^{-4}$ is applied to prevent overfitting. The training process is executed on 8 NVIDIA A100 40GB GPUs, leveraging their computational power to handle the substantial model and dataset.

During the prediction phase for binding affinity, we adapt our model to a 4-layer Transformer configuration. In this stage, we reduce the batch size to 8, optimizing for lower memory consumption while maintaining effective learning. The learning rate is adjusted to $3 \times 10^{-4}$, ensuring adequate convergence. This phase is executed on 2 NVIDIA A100 40GB GPUs, enabling efficient processing of predictions while preserving computational resources.

### Ablation study

To validate the effectiveness of each key component in our model and its contribution to overall performance, we conduct a series of detailed ablation studies. First, we investigate the impact of disabling distance information, spatial positional information, and structural information (with both distance and spatial positional information disabled) on model performance. As shown in Table 5, the results demonstrate that structural information is crucial for the model, as it provides essential spatial constraints for accurate predictions. Additionally, we examine the model’s performance in scenarios without ligand pre-training and without any pre-training of both proteins and ligands.  The results in Table 5 indicate the absence of ligand pre-training results in a significant drop in model performance, underscoring the effectiveness of the small molecule pre-training approach. Furthermore, when no pre-training is applied at all, the model’s performance deteriorates even further, highlighting the importance of pre-training in enhancing model capabilities. Pre-training facilitates better initialization parameters, accelerates convergence, and improves the model’s generalization ability. These ablation experiments clearly illustrate that each critical component is indispensable within the model. Distance information, spatial positional information, structural information, and pre-training all contribute significantly to the model’s performance. The synergistic interaction of these components enables our model to achieve outstanding results in binding affinity prediction.

**Table 5:** Ablation Study Results on the LBA30 Dataset, results in bold indicate the best performance for each column

Model configuration	RMSE	Pearson	Spearman
Disabled distance information	**1.522**	0.454	0.455
Disabled spatial position information	1.577	0.445	0.438
Disabled structure information	2.088	0.435	0.430
w/o Ligand pre-trained model	1.578	0.453	0.435
w/o pre-trained model	1.639	0.390	0.420
SableBind	1.527	**0.579**	**0.579**

**Fig. 4:**
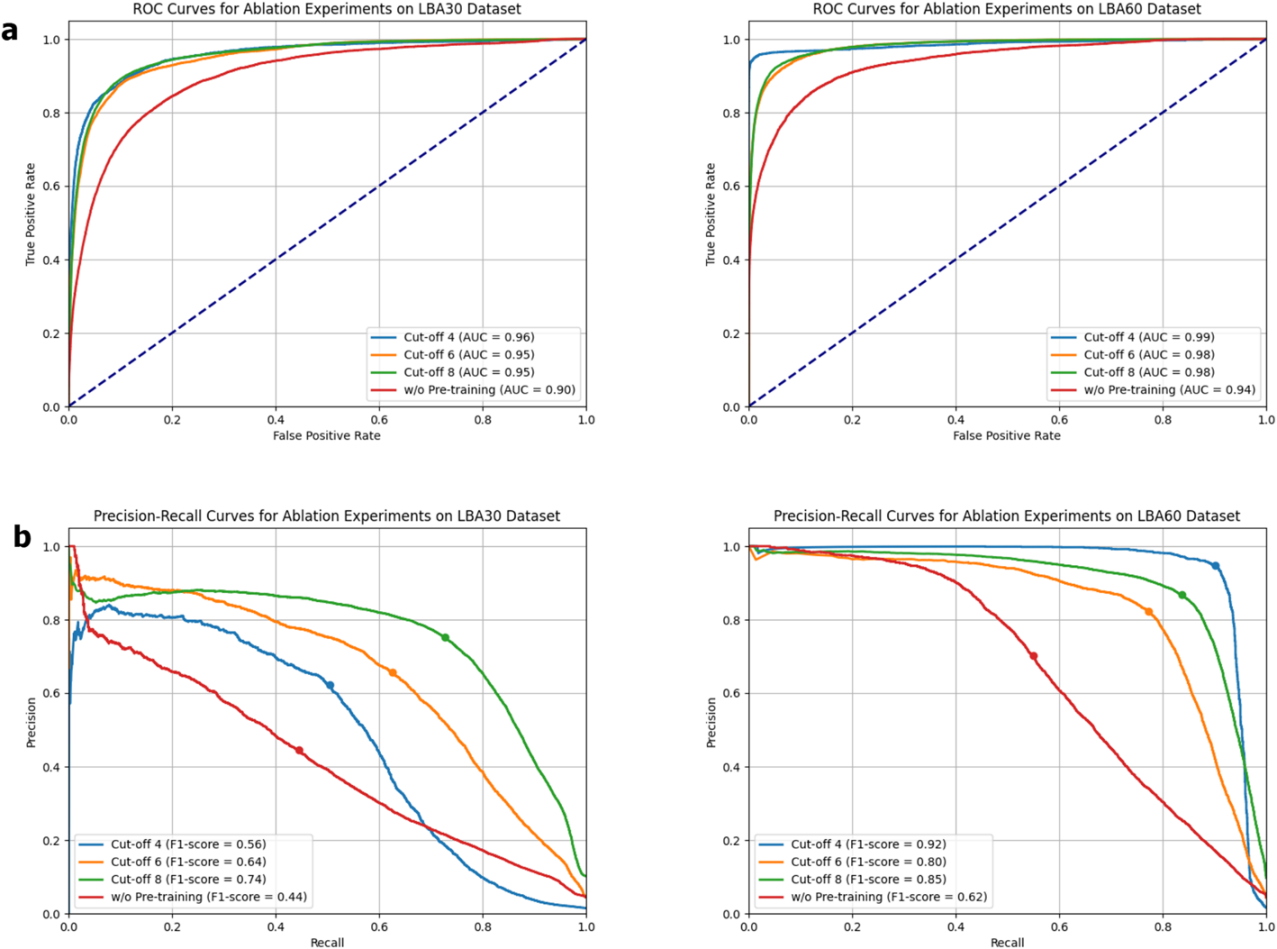
Ablation experiments for binding site prediction under different cut-offs and without pre-training models. Panel **a** illustrates the ROC curves for the LBA30 (left) and LBA60 (right) datasets, while panel **b** displays the corresponding PR curves. The “cut-off” refers to the predefined distance threshold for defining binding site residues, with values of 4Å, 6Å, and 8Åused in the experiments

Furthermore, we conduct an ablation experiment on protein-ligand binding site prediction. As illustrated in Fig. 4, the ROC curves for the LBA30 and LBA60 datasets indicate that varying cut-off values have a relatively minor impact. This suggests that, within the examined range, changes in the threshold do not significantly alter the relationship between the true positive rate and the false positive rate. In contrast, the PR curves show more pronounced differences, highlighting that the precision-recall trade-off is more sensitive to threshold variations. This discrepancy likely arises because ROC curves primarily focus on the overall discrimination capability of the model, while PR curves are more attuned to the balance between precision and recall. Consequently, different thresholds can affect the model’s ability to accurately classify positive and negative examples, leading to more substantial shifts in precision and recall.

Moreover, when comparing the results obtained without pre-training (using a predefined threshold of 6 angstroms) to those with pre-training, we observe a significant drop in performance. This clearly underscores the importance of our pre-training model, which provides crucial support for enhancing performance and generalization in binding site prediction. Notably, across all scenarios, our model consistently demonstrates high classification performance, as evidenced by the overall trends in both the ROC and PR curves. Despite variations due to different thresholds, the model maintains robustness in distinguishing between positive and negative cases.

## Conclusion

In this research paper, we present a novel method for predicting protein-ligand binding affinity based on pre-trained models. By integrating distance and spatial position information of ligands, our approach successfully predicts binding affinity values without prior knowledge of specific binding pocket information on the protein. Compared to benchmark baselines, our method demonstrates superior correlation, highlighting its effectiveness in capturing the complexities of protein-ligand interactions.

The ablation experiments reveal that, without pre-training, various performance indicators of the model decline significantly, underscoring the crucial role of pre-training in enhancing overall performance. The pre-trained model developed for ligands holds great promise for wide-ranging applications in molecular structure representation learning and can be utilized for various downstream tasks, such as predicting molecular properties.

Looking ahead, our method is poised for further extension, enabling not only the prediction of binding affinity but also the accurate determination of protein-ligand complex structures through pair representation. This advancement broadens the applicability of our approach across diverse contexts. More importantly, we aim to unify the representations of biomolecules to dismantle the barriers between different biomolecular representations. By establishing a unified methodology for cross-molecular scale pre-training, we can simultaneously capture interactions among diverse biomolecules and predict the binding of multiple biomolecules, thereby enhancing the model’s generalization capabilities.

## Data Availability

The source code and data sets are available at https://github.com/MIALAB-RUC/SableBind.

## Declarations

### Compiting interest

The authors declare that they have no compiting interest.

## References

[1] Cohen P. Protein kinases-the major drug targets of the twenty-first century? Nat Rev Drug Discov. 2002;1(4):309–15. 12120282 10.1038/nrd773

[2] Noble ME, Endicott JA, Johnson LN. Protein kinase inhibitors: insights into drug design from structure. Science. 2004;303(5665):1800–5. 15031492 10.1126/science.1095920

[3] Hansch C, Fujita T. p-- analysis. a method for the correlation of biological activity and chemical structure. J Am Chem Soc. 1964;86(8):1616–26.

[4] Tropsha A, Isayev O, Varnek A, Schneider G, Cherkasov A. Integrating QSAR modelling and deep learning in drug discovery: the emergence of deep QSAR. Nat Rev Drug Discov. 2024;23(2):141–55. 38066301 10.1038/s41573-023-00832-0

[5] Kitchen DB, Decornez H, Furr JR, Bajorath J. Docking and scoring in virtual screening for drug discovery: methods and applications. Nat Rev Drug Discov. 2004;3(11):935–49. 15520816 10.1038/nrd1549

[6] Wang R, Lai L, Wang S. Further development and validation of empirical scoring functions for structure-based binding affinity prediction. J Comput Aided Mol Des. 2002;16:11–26. 12197663 10.1023/a:1016357811882

[7] Friesner RA, Banks JL, Murphy RB, Halgren TA, Klicic JJ, Mainz DT, Repasky MP, Knoll EH, Shelley M, Perry JK, et al. Glide: a new approach for rapid, accurate docking and scoring. 1. method and assessment of docking accuracy. J Med Chem. 2004;47(7):1739–49. 15027865 10.1021/jm0306430

[8] Hollingsworth SA, Dror RO. Molecular dynamics simulation for all. Neuron. 2018;99(6):1129–43. 30236283 10.1016/j.neuron.2018.08.011 PMC6209097

[9] Jiménez J, Skalic M, Martinez-Rosell G, De Fabritiis G. K deep: protein-ligand absolute binding affinity prediction via 3d-convolutional neural networks. J Chem Inf Model. 2018;58(2):287–96. 29309725 10.1021/acs.jcim.7b00650

[10] Stepniewska-Dziubinska MM, Zielenkiewicz P, Siedlecki P. Development and evaluation of a deep learning model for protein-ligand binding affinity prediction. Bioinformatics. 2018;34(21):3666–74. 29757353 10.1093/bioinformatics/bty374 PMC6198856

[11] Hassan-Harrirou H, Zhang C, Lemmin T. Rosenet: improving binding affinity prediction by leveraging molecular mechanics energies with an ensemble of 3d convolutional neural networks. J Chem Inf Model. 2020;60(6):2791–802. 32392050 10.1021/acs.jcim.0c00075

[12] Feinberg EN, Sur D, Wu Z, Husic BE, Mai H, Li Y, Sun S, Yang J, Ramsundar B, Pande VS. Potentialnet for molecular property prediction. ACS Cent Sci. 2018;4(11):1520–30. 30555904 10.1021/acscentsci.8b00507 PMC6276035

[13] Shen C, Zhang X, Deng Y, Gao J, Wang D, Xu L, Pan P, Hou T, Kang Y. Boosting protein-ligand binding pose prediction and virtual screening based on residue-atom distance likelihood potential and graph transformer. J Med Chem. 2022;65(15):10691–706. 35917397 10.1021/acs.jmedchem.2c00991

[14] Moon S, Zhung W, Yang S, Lim J, Kim WY. Pignet: a physics-informed deep learning model toward generalized drug-target interaction predictions. Chem Sci. 2022;13(13):3661–73. 35432900 10.1039/d1sc06946b PMC8966633

[15] Xu Z, Wang S, Zhu F, Huang J (2017) Seq2seq fingerprint: an unsupervised deep molecular embedding for drug discovery. In: Proceedings of the 8th ACM international conference on bioinformatics, computational biology, and health informatics, pp 285–294

[16] Wang S, Guo Y, Wang Y, Sun H, Huang J (2019) Smiles-bert: large scale unsupervised pre-training for molecular property prediction. In: Proceedings of the 10th ACM international conference on bioinformatics, computational biology and health informatics, pp 429–436

[17] Winter R, Montanari F, Noé F, Clevert D-A. Learning continuous and data-driven molecular descriptors by translating equivalent chemical representations. Chem Sci. 2019;10(6):1692–701. 30842833 10.1039/c8sc04175j PMC6368215

[18] Hu W, Liu B, Gomes J, Zitnik M, Liang P, Pande V, Leskovec J (2019) Strategies for pre-training graph neural networks. arXiv preprint arXiv:1905.12265

[19] Li P, Wang J, Qiao Y, Chen H, Yu Y, Yao X, Gao P, Xie G, Song S. An effective self-supervised framework for learning expressive molecular global representations to drug discovery. Brief Bioinform. 2021;22(6):109. 33940598 10.1093/bib/bbab109

[20] Wu F, Jin S, Jiang Y, Jin X, Tang B, Niu Z, Liu X, Zhang Q, Zeng X, Li SZ. Pre-training of equivariant graph matching networks with conformation flexibility for drug binding. Adv Sci. 2022;9(33):2203796. 10.1002/advs.202203796 PMC9685463 36202759

[21] Rong Y, Bian Y, Xu T, Xie W, Wei Y, Huang W, Huang J. Self-supervised graph transformer on large-scale molecular data. Adv Neural Inf Process Syst. 2020;33:12559–71.

[22] Wang Y, Wang J, Cao Z, Barati Farimani A. Molecular contrastive learning of representations via graph neural networks. Nat Mach Intell. 2022;4(3):279–87.

[23] Fang X, Liu L, Lei J, He D, Zhang S, Zhou J, Wang F, Wu H, Wang H. Geometry-enhanced molecular representation learning for property prediction. Nat Mach Intell. 2022;4(2):127–34.

[24] Luo Y, Ji S (2022) An autoregressive flow model for 3d molecular geometry generation from scratch. In: International conference on learning representations (ICLR) 2022

[25] Jing B, Corso G, Chang J, Barzilay R, Jaakkola T. Torsional diffusion for molecular conformer generation. Adv Neural Inf Process Syst. 2022;35:24240–53.

[26] Rudrapal M, Chetia D. Virtual screening, molecular docking and QSAR studies in drug discovery and development programme. J Drug Deliv Ther. 2020;10(4):225–33.

[27] Zhou G, Gao Z, Ding Q, Zheng H, Xu H, Wei Z, Zhang L, Ke G (2023) Uni-mol: a universal 3d molecular representation learning framework. In: The Eleventh International Conference on Learning Representations 2023. https://openreview.net/forum?id=6K2RM6wVqKu

[28] Feng S, Li M, Jia Y, Ma W, Lan Y (2023) Protein-ligand binding representation learning from fine-grained interactions. arXiv preprint arXiv:2311.16160

[29] Ni Y, Feng S, Hong X, Sun Y, Ma W-Y, Ma Z-M, Ye Q, Lan Y. Pre-training with fractional denoising to enhance molecular property prediction. Nat Mach Intell. 2024;2024:1–10.

[30] Ye Q, Li J, Chen X, Huang H, Zeng M, Yu J, Gong X (2024) Sable: bridging the gap in protein structure understanding with an empowering and versatile pre-training paradigm. PREPRINT (Version 1) available at Research Square [10.21203/rs.3.rs-4647798/v1] 10.1093/bib/bbaf120 PMC11957296 40163822

[31] Wang R, Fang X, Lu Y, Wang S. The pdbbind database: collection of binding affinities for protein- ligand complexes with known three-dimensional structures. J Med Chem. 2004;47(12):2977–80. 15163179 10.1021/jm030580l

[32] Su M, Yang Q, Du Y, Feng G, Liu Z, Li Y, Wang R. Comparative assessment of scoring functions: the CASF-2016 update. J Chem Inf Model. 2018;59(2):895–913. 30481020 10.1021/acs.jcim.8b00545

[33] Townshend RJ, Vögele M, Suriana P, Derry A, Powers A, Laloudakis Y, Balachandar S, Jing B, Anderson B, Eismann S, et al. (2020) Atom3d: tasks on molecules in three dimensions. arXiv preprint arXiv:2012.04035

[34] Öztürk H, Özgür A, Ozkirimli E. Deepdta: deep drug-target binding affinity prediction. Bioinformatics. 2018;34(17):821–9. 10.1093/bioinformatics/bty593 PMC6129291 30423097

[35] Rao R, Bhattacharya N, Thomas N, Duan Y, Chen P, Canny J, Abbeel P, Song Y. Evaluating protein transfer learning with tape. Adv Neural Inf Process Syst. 2019;32(869):7112–27. PMC7774645 33390682

[36] Elnaggar A, Heinzinger M, Dallago C, Rehawi G, Wang Y, Jones L, Gibbs T, Feher T, Angerer C, Steinegger M, et al. Prottrans: toward understanding the language of life through self-supervised learning. IEEE Trans Pattern Anal Mach Intell. 2021;44(10):7112–27. 10.1109/TPAMI.2021.3095381 34232869

[37] Somnath VR, Bunne C, Krause A. Multi-scale representation learning on proteins. Adv Neural Inf Process Syst. 2021;34:25244–55.

[38] Wang L, Liu H, Liu Y, Kurtin J, Ji S (2022) Learning hierarchical protein representations via complete 3d graph networks. arXiv preprint arXiv:2207.12600

[39] Karimi M, Wu D, Wang Z, Shen Y. Deepaffinity: interpretable deep learning of compound-protein affinity through unified recurrent and convolutional neural networks. Bioinformatics. 2019;35(18):3329–38. 30768156 10.1093/bioinformatics/btz111 PMC6748780

[40] Pei Q, Wu L, Zhu J, Xia Y, Xie S, Qin T, Liu H, Liu T-Y (2022) SMT-DTA: improving drug-target affinity prediction with semi-supervised multi-task training. arXiv preprint arXiv:2206.09818

[41] Liu S, Guo H, Tang J (2022) Molecular geometry pretraining with se (3)-invariant denoising distance matching. arXiv preprint arXiv:2206.13602

[42] Trott O, Olson AJ. Autodock vina: improving the speed and accuracy of docking with a new scoring function, efficient optimization, and multithreading. J Comput Chem. 2010;31(2):455–61. 19499576 10.1002/jcc.21334 PMC3041641

[43] Friesner RA, Murphy RB, Repasky MP, Frye LL, Greenwood JR, Halgren TA, Sanschagrin PC, Mainz DT. Extra precision glide: docking and scoring incorporating a model of hydrophobic enclosure for protein- ligand complexes. J Med Chem. 2006;49(21):6177–96. 17034125 10.1021/jm051256o

[44] Sánchez-Cruz N, Medina-Franco JL, Mestres J, Barril X. Extended connectivity interaction features: improving binding affinity prediction through chemical description. Bioinformatics. 2021;37(10):1376–82. 33226061 10.1093/bioinformatics/btaa982

[45] Jin Z, Wu T, Chen T, Pan D, Wang X, Xie J, Quan L, Lyu Q. Capla: improved prediction of protein-ligand binding affinity by a deep learning approach based on a cross-attention mechanism. Bioinformatics. 2023;39(2):049. 10.1093/bioinformatics/btad049 PMC9900214 36688724

[46] Shen L, Feng H, Qiu Y, Wei G-W. SVSBI: sequence-based virtual screening of biomolecular interactions. Commun Biol. 2023;6(1):536. 37202415 10.1038/s42003-023-04866-3 PMC10195826

[47] Wang Z, Zheng L, Liu Y, Qu Y, Li Y-Q, Zhao M, Mu Y, Li W. Onionnet-2: a convolutional neural network model for predicting protein-ligand binding affinity based on residue-atom contacting shells. Front Chem. 2021;9: 753002. 34778208 10.3389/fchem.2021.753002 PMC8579074

[48] Shen C, Zhang X, Hsieh C-Y, Deng Y, Wang D, Xu L, Wu J, Li D, Kang Y, Hou T, et al. A generalized protein-ligand scoring framework with balanced scoring, docking, ranking and screening powers. Chem Sci. 2023;14(30):8129–46. 37538816 10.1039/d3sc02044d PMC10395315

[49] Luo D, Liu D, Qu X, Dong L, Wang B. Enhancing generalizability in protein-ligand binding affinity prediction with multimodal contrastive learning. J Chem Inf Model. 2024;64(6):1892–906. 38441880 10.1021/acs.jcim.3c01961

[50] Moon S, Hwang S-Y, Lim J, Kim WY. Pignet2: a versatile deep learning-based protein-ligand interaction prediction model for binding affinity scoring and virtual screening. Digital Discov. 2024;3(2):287–99.

[51] Chen D, Liu J, Wei G-W. Multiscale topology-enabled structure-to-sequence transformer for protein-ligand interaction predictions. Nat Mach Intell. 2024;6(7):799–810. 10.1038/s42256-024-00855-1 PMC12290916 40718138

[52] Morris GM, Huey R, Lindstrom W, Sanner MF, Belew RK, Goodsell DS, Olson AJ. Autodock4 and autodocktools4: automated docking with selective receptor flexibility. J Comput Chem. 2009;30(16):2785–91. 19399780 10.1002/jcc.21256 PMC2760638

[53] Quiroga R, Villarreal MA. Vinardo: a scoring function based on autodock vina improves scoring, docking, and virtual screening. PLoS ONE. 2016;11(5):0155183. 10.1371/journal.pone.0155183 PMC4865195 27171006

[54] Schütt KT, Arbabzadah F, Chmiela S, Müller KR, Tkatchenko A. Quantum-chemical insights from deep tensor neural networks. Nat Commun. 2017;8(1):13890. 28067221 10.1038/ncomms13890 PMC5228054

[55] Lim J, Ryu S, Park K, Choe YJ, Ham J, Kim WY. Predicting drug-target interaction using a novel graph neural network with 3d structure-embedded graph representation. J Chem Inf Model. 2019;59(9):3981–8. 31443612 10.1021/acs.jcim.9b00387

[56] Jiang D, Hsieh C-Y, Wu Z, Kang Y, Wang J, Wang E, Liao B, Shen C, Xu L, Wu J, et al. Interactiongraphnet: a novel and efficient deep graph representation learning framework for accurate protein-ligand interaction predictions. J Med Chem. 2021;64(24):18209–32. 34878785 10.1021/acs.jmedchem.1c01830

[57] Yang C, Zhang Y. Delta machine learning to improve scoring-ranking-screening performances of protein-ligand scoring functions. J Chem Inf Model. 2022;62(11):2696–712. 35579568 10.1021/acs.jcim.2c00485 PMC9197983

[58] Yang Z, Zhong W, Lv Q, Dong T, Yu-Chian Chen C. Geometric interaction graph neural network for predicting protein-ligand binding affinities from 3d structures (GIGN). J Phys Chem Lett. 2023;14(8):2020–33. 36794930 10.1021/acs.jpclett.2c03906

[59] Gabel J, Desaphy J, Rognan D. Beware of machine learning-based scoring functions-on the danger of developing black boxes. J Chem Inf Model. 2014;54(10):2807–15. 25207678 10.1021/ci500406k

[60] Li Y, Yang J. Structural and sequence similarity makes a significant impact on machine-learning-based scoring functions for protein-ligand interactions. J Chem Inf Model. 2017;57(4):1007–12. 28358210 10.1021/acs.jcim.7b00049

